# Properties of Particulate Matter in the Air of the Wieliczka Salt Mine and Related Health Benefits for Tourists

**DOI:** 10.3390/ijerph19020826

**Published:** 2022-01-12

**Authors:** Karolina Bralewska, Wioletta Rogula-Kozłowska, Dominika Mucha, Artur Jerzy Badyda, Magdalena Kostrzon, Adrian Bralewski, Stanisław Biedugnis

**Affiliations:** 1 Safety Engineering Institute, The Main School of Fire Service, Slowackiego Street, 52/54, 01-629 Warsaw, Poland; wrogula@sgsp.edu.pl (W.R.-K.); sbiedugnis@sgsp.edu.pl (S.B.); 2 Faculty of Building Services, Hydro and Environmental Engineering, Warsaw University of Technology, Nowowiejska Street, 20, 00-653 Warsaw, Poland; dominika.mucha@pw.edu.pl (D.M.); artur.badyda@pw.edu.pl (A.J.B.); 3 Wieliczka Salt Mine Health Resort, Park Kingi Street, 1, 32-020 Wieliczka, Poland; uzdrowisko@kopalnia.pl; 4 Faculty of Safety Engineering and Civil Protection, The Main School of Fire Service, Slowackiego Street, 52/54, 01-629 Warsaw, Poland; abralewski@sgsp.edu.pl

**Keywords:** particulate matter, salt aerosol, salt mine microclimate, particle mass size distribution, health effects, health resort

## Abstract

This study aimed to evaluate the mass concentration of size-resolved (PM_1_, PM_2.5_, PM_4_, PM_10_, PM_100_) particulate matter (PM) in the Wieliczka Salt Mine located in southern Poland, compare them with the concentrations of the same PM fractions in the atmospheric air, and estimate the dose of dry salt aerosol inhaled by the mine visitors. Measurements were conducted for 2 h a day, simultaneously inside (tourist route, passage to the health resort, health resort) and outside the mine (duty-room), for three days in the summer of 2017 using DustTrak DRX devices (optical method). The highest average PM concentrations were recorded on the tourist route (54–81 µg/m^3^), while the lowest was in the passage to the health resort (49–62 µg/m^3^). At the same time, the mean outdoor PM concentrations were 14–20 µg/m^3^. Fine particles constituting the majority of PM mass (68–80%) in the mine originated from internal sources, while the presence of coarse particles was associated with tourist traffic. High PM deposition factors in the respiratory tract of children and adults estimated for particular mine chambers (0.58–0.70), the predominance of respirable particles in PM mass, and the high content of NaCl in PM composition indicate high health benefits for mine visitors.

## 1. Introduction

Research conducted by [1,2,3,4,5,6] proves that the microclimate of underground salt chambers and therapy in such places have beneficial effects on the respiratory system and may even cause gradual restoration of health and functional efficiency of many physiological systems. One of the treatment methods belonging to the climatotherapeutic methods is subterraneotherapy. This method is based on repeated and long-term exposure of patients to the specific influence of intense physical, chemical and biological stimuli occurring in the underground chambers or caves created during excavations in salt mining [7]. Indications for treatment with this method include the upper and lower respiratory tract diseases, with particular emphasis on chronic obstructive pulmonary disease (COPD), recurrent rhinitis and inflammations of the sinuses, throat, larynx, chronic bronchitis, pneumonia, allergic diseases and bronchial asthma [7].

Due to the strict requirements for air quality and microclimate parameters, subterraneotherapy is implemented only in a few centres around the world, including the Salt Mines in Wieliczka and Bochnia (Poland), in Turda (Romania), in Zlote Hory (Czech Republic) and Berchtesgaden (Germany) [3]. The most important elements of the microclimate determining the therapeutic nature of mine workings are: increased (in relation to the values on the surface of the earth) air pressure, high relative humidity (60–80%), constant low temperature (13–14.5 °C), high degree of air ionisation, low content of bacteria and fungi and isolation from environmental pollutants, including particulate matter inflowing from the surroundings [8,9,10,11,12,13]. After entering the underground space, the air undergoes physicochemical and biological changes, both in terms of quality and quantity. This means that some components are reduced in volume (e.g., oxygen), while others are increased (e.g., carbon dioxide) or new ones appear, e.g., radon, methane, hydrogen sulphide and sodium chloride, calcium or magnesium aerosol [14]. Mine aerosols are two or three-component systems, in which the medium (dispersion phase) is changed air, and the component (dispersed phase) is solid (grain) or liquid (liquid particles) which due to the high degree of ionisation are charged with an electric charge, most often negative.

The mine aerosol includes, in particular, sodium chloride (NaCl), which is highly soluble in water, mainly in a dissociated state [3,9,10,15]. The concentration of NaCl in underground chambers may reach several dozen milligrams per cubic meter of air [11]. According to the previous studies, NaCl constitutes more than 80% of PM mass in the chambers of the salt mine in Wieliczka [10,14,16]. The presence of this compound in the air of salt mines is related to the constantly occurring dissolution of salt rocks by humid air, saline stagnant waters, brine springs and watercourses. In addition to the ingredients beneficial to human health, the mine aerosol also contains organic and inorganic compounds considered as air pollutants. Mainly, it is material transported underground along with the air coming from the external environment through a ventilation tunnel or related to the presence and activity of people and equipment in the mine [10,15].

One of the important factors in the mine’s microclimate is particulate matter (PM). Previous studies on PM mainly focus on its negative impact on human health [17,18,19,20,21,22]. Many studies have shown that the impact of PM on human health depends mainly on the size of the particles, their ability to migrate through the respiratory tract and the content of toxic components [23,24,25]. In some places, such as underground health resorts, salt caves, brine graduation towers, due to the high content of chlorine, sodium, magnesium and calcium ions, suspended solid particles may constitute also a therapeutic factor [8,10,15,26,27]. The physical and chemical properties of a dry aerosol are different from that of a wet aerosol, and the dry aerosol is more effective in treatment [26].

The studies described in this work consisted of simultaneously determining the PM concentration in the underground chamber complex of the Wieliczka Salt Mine and its external environment. The mass concentration of size-resolved (PM_1_, PM_1-2.5_, PM_2.5-4_, PM_4-10_, PM_10-100_) total suspended particles (TSP) were determined using the optical method. The objectives of this study were to determine the differences in the concentrations of PM in individual areas of the mine (tourist route, passage to the health resort, health resort), to compare PM concentrations and its size composition inside and outside the salt mine, and to estimate the dose of dry NaCl inhaled by tourists visiting the mine and patients using the health resort services. A review of the literature on the subject indicates that this is one of the few works in which the concentrations of PM in the air of underground spaces were recognised. So far, studies of PM concentrations in rehabilitation rooms located in mining excavation were carried out only by [9,10,15]. This work is a continuation of the research by Rogula-Kozłowska and Badyda with their teams, carried out in the same mine in 2015 [10].

## 2. Materials and Methods

### 2.1. Sampling Site

**Figure 1:**
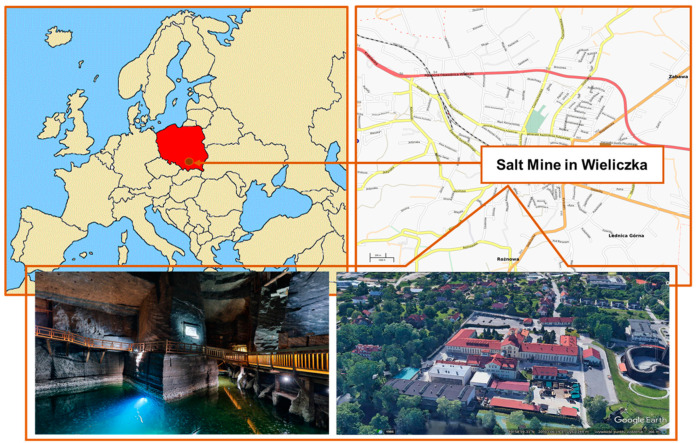
Sampling site.

Wieliczka Salt Mine Health Resort is located in the southern part of Poland (49°58′59.37″ N, 20°03′05.85″ E) in the Małopolska Region (Figure 1), which is often defined as the most polluted region of the country [28,29,30]. The immediate vicinity of the mine (within a radius of 3 km) is characterised by single-family houses. The mine is located 3 km (in a straight line) south of the A4 motorway. The main source of air pollutants in the vicinity of the mine is the surface emission related to individual heating of buildings in the municipal and households sector (low-stack emissions), as well as traffic emissions [31]. Until 1996, the salt mine in Wieliczka was an active mining facility for 700 years. Currently, it is a historical facility with the status of a health resort. It is a UNESCO World Heritage Site. More than a million people come to it annually for tourist purposes, of which approx. 2.2% are people visiting the health resort of the mine [32].

### 2.2. Sampling Method

**Figure 2:**
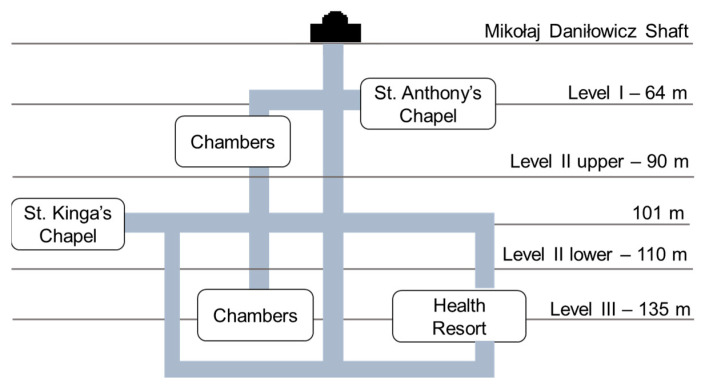
A simplified schematic map of the Wieliczka Salt Mine.

Three two-hour measurements were carried out at the turn of July and August 2017 (7 November 2017 between 3 p.m. and 5 p.m.; 7 December 2017 between 9 a.m. and 11 a.m.; 8 March 2017 between 2 p.m–4 p.m.). The measurements consisted of measuring the mass concentrations of five PM fractions (PM_1_, PM_2.5_, PM_4_, PM_10_, TSP (alternatively referred to as PM_100_)) simultaneously inside and outside the mine. Inside the mine, measurements were conducted at depths in the range of 0–135 meters underground (Figure 2). First, measurements were carried out along the entire tourist route (entrance from the Daniłowicz Shaft, Chambers: Nicolaus Copernicus, St Anthony’s Chapel, Janowice, Spalone, Casimir the Great, Pieskowa Skała, St Kinga’s Chapel, Michałowice, Weimar, Stanisław Staszic, Fortymbark, Gospoda, Boczaniec, Franciszek Karol), then (without any break) along the route leading to the health resort, and finally in the health resort chambers (Smok Chamber, Wessel Lake Chamber, Boczkowski Chamber, Eastern Mountains’ Stable Chamber) [33]. At each measuring point, the meter was at a height of 1 m with 1 m free space around the device. Outside, the device was set on an external window sill of the duty station (entrance to the mine) 1.5 m from the ground surface on the west side of the building. Two DustTrak DRX 8534 meters were used for measurements (one inside, one outside the mine). The devices recorded the results with a 1 s time resolution. DustTrak allows for simultaneous real-time mass concentration measurements of PM with aerodynamic diameters of 0.1–100 μm in the range of 0.001–150 mg/m^3^. The accuracy of the sampler is 5%. Aerosol being measured is drawn into the optical chamber in a continuous stream using a pump at a total flow rate of 3 L/min. The devices were calibrated before the measurements (7 March 2017) using a standardised dust sample (Arizona Dust; TSI). Zero calibration was performed using a high-efficiency particulate air (HEPA) filter before each measurement.

Because optical meters do not use a reference measurement method and because in cases of high air humidity the optical devices overestimate the results [34,35,36,37] equivalence tests were conducted before the measurements according to European Commission Guidance for the Demonstration of Equivalence of Ambient Air Monitoring Methods [38]. The tests consisted of parallel measurements of PM_2.5_ and TSP concentrations using the reference and non-reference methods. For this purpose, forty 8 h PM_2.5_ and TSP concentration measurements using the gravimetric (GilAir3 aspirators) and optical method (DustTrak 8534) were conducted. Calculations of the linear regression coefficients a and b for each set of measurements were made. Differences between PM concentrations measured by the gravimetric and the optical methods were lower than 25%; slope coefficient “b” took values in the range of 1.0–1.4 and coefficient “a” (the point of intersection of the regression line with the *X*-axis) took values 0.1–2.6. Coefficients of determination (R^2^) took values in the range of 0.95–0.97. The results obtained by the DustTrak devices were reduced by the average value of the “a” coefficient (defined separately for fine (1–2.5 µm) and coarse particles (2.5–100 µm)).

### 2.3. Result Analysis

The PM mass concentrations of five PM fractions (PM_1_, PM_2.5_, PM_4_, PM_10_, PM_100_) inside (tourist route, passage to the health resort, health resort) and outside the mine were used to determine basic characteristics of PM, i.e., average, minimum, maximum and central (median) concentrations, as well as standard deviation from the mean. The measured PM concentrations were also used to compare the PM mass size distribution and to analyse the variability of concentrations along the tourist route, the passage to the health resort and in the health resort. The mass median aerodynamic diameter (MMAD) and the geometric standard deviation (GSD), two parameters characterising the particle size distribution (PSD), were determined using regression lines from the log-probability graph of PM size versus cumulative mass distribution [39]. If d_p_ denotes the diameter for which p% of the total mass of particles are smaller than d_p_, then MMAD for a PSD is d_50_. The GSD is computed as the ratio d_84.1_/d_50_ = d_50_/d_15.9_ for unimodal PSDs and as square root of the ratio d_84.1_/d_15.9_ for bimodal PSDs [39,40].

Based on the MMAD and GSD values estimated for individual locations (tourist route, passage to the health resort, health resort), PM deposition factors were calculated for three anatomically separate airway regions (RT), i.e., upper respiratory tract (H), trachea and bronchi (TB), and alveoli (P). The deposition factors were calculated using the Multiple Path Particle Dosimetry (MPPD v. 3.04, Ara Inc.) model developed by the Chemical Industry Institute of Chemical Toxicology and the Dutch National Institute of Public Health and the Environment. An age-specific symmetric lung model was adopted for modelling purposes. Two age groups were taken into account in the calculations - adults over the age of 21 and children over the age of 8. The deposition factor was calculated for one breathing cycle including only inspiration (without expiration). The following scenario was considered: upright body orientation, nasal breathing under conditions of normal physiological activity. Specific physiological parameters used in calculations are summarised in Table 1 [41]. Then, using the methodology developed by the U.S. Environmental Protection Agency (US EPA) and based on estimated PM deposition factors, the average doses of respirable particles of salt aerosol inhaled at individual points of the mine (D_i_) by adults (21+) and children (8+) were calculated [42,43,44,45]. Based on previous studies, for the purposes of the calculations, it was assumed that sodium chloride (NaCl) accounts for 88% of PM mass on average [10,14,16]. The following formula was used for calculations:

D_i_ = DF_i_ × C_i_ × t_i_ × InhR, (1)

where:

D_i_ is the average dose of inhaled aerosol during the stay at the i-th measurement point (tourist route/passage to the health resort/health resort) [µg],
DF_i_ is the deposition factor at the i-th measuring point,
C_i_ is the average concentration of respirable fraction PM (PM_4_) at the i-th measurement point [µg/m^3^],
t_i_ is the average time of stay at the i-th measurement point [day],
InhR is inhalation rate (m^3^/day).

## 3. Results

### 3.1. Distribution of Particulate Matter Concentrations in the Mine

**Figure 3:**
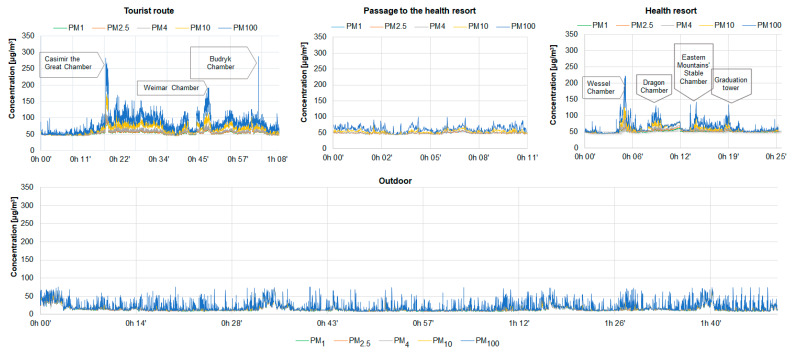
Distribution of concentrations of five PM fractions on the tourist route, in the passage to the health resort, in the health resort and outside the mine.

The statistical parameters of five PM fractions (PM_1_, PM_2.5_, PM_4_, PM_10_ and PM_100_) at various points in the Wieliczka salt mine, i.e., along the tourist route, in the passage to the health resort and the health resort are presented in Table 2. PM concentrations inside the mine fluctuated depending on the location of the measuring point. The lowest average concentrations of each of the five PM fractions were recorded in the passage to the health resort (48.9 µg/m^3^ for PM_1_, 61.7 µg/m^3^ for PM_100_), while the highest on the tourist route (54.5 µg/m^3^ for PM_1_, 81.2 µg/m^3^ for PM_100_). The concentrations changed most dynamically on the tourist route and in the health resort, where at some measurement points the concentrations of PM_100_ reached maximum values above 200 µg/m^3^ (Figure 3). The relatively high fluctuations in concentrations along the tourist route are also evidenced by the highest standard deviations (Table 2). The lowest standard deviations are estimated for the concentrations measured in the passage to the health resort. Considering the deviations for individual PM fractions, it was found that in all locations the highest standard deviations were recorded for PM_100_ (Table 2). The factors that could have influenced the fluctuations in PM concentrations along the tourist route, and at the same time the source of coarse PM, i.e., particles with aerodynamic diameters greater than 2.5 µm, could be tourist traffic, and more precisely, life processes and pollution brought by tourists or mine workers (clothing, flaky skin, hair, food), also activities related to the maintenance of the mine, e.g., repairs in chambers excluded from tourist traffic. In the case of the health resort, the highest concentration fluctuations, especially of coarse PM, were observed in rooms where gymnastic classes or medical treatments were held (Wessel Chamber (6′) and Dragon Chamber (17′)). In each of the individual health resort points marked in Figure 3, there were at the same time less than 15 people. The obtained results allow one to conclude that the fluctuations in PM concentrations in the health resort, and at the same time the presence of coarse PM in this place was determined by the activity of patients, the use of rehabilitation devices or the use of various types of cosmetics [10,15,46]. It should also be added that during the holiday months, i.e., when the measurements were taken, the intensity of tourist traffic in the mine is the highest (3000–3600 people per day), while the number of visits in the health resort is the lowest (30–45 people per day) [32]. The assumptions about the influence of tourist traffic on fluctuations in PM concentrations and the origin of coarse PM are also confirmed by the observations made during the research. Large, organised groups of tourists, e.g., trips of school children of 15–30 people, were passed at points corresponding to fluctuations in PM concentrations (Casimir the Great Chamber (18–20′), Weimar Chamber (47–49′), especially PM_10_ and PM_100_. Budryk Chamber (1:04′) is a restaurant where about a hundred people were staying at the same time. In addition, an increase in PM concentrations was also observed in points with relatively large spaces and places with large rock walls, e.g., Sielec Chamber (17′), Pieskowa Skała Chamber (22–25′), St. Kinga Chapel (28′) and the Erazm Barącz Chamber with an underground lake (37′). Those fluctuations of both fine and coarse PM concentrations were observed at these locations. This could be due to the increase in the proportion of mineral salts and aerosols in these places. Although the passage to the health resort and the corridors between individual chambers are low and narrow (approx. 2.5 m × 2 m), PM concentrations were the lowest at these measuring points. It was probably related to the intense air circulation and strong drafts in these places. As many researchers consider the median to be a better representative of measure than the arithmetic mean, both values are summarised in Table 2. Often the arithmetic mean is overestimated by dynamic concentration fluctuations and associated with the maximum concentration. In the present case, the medians were actually lower than the average concentrations. The biggest differences are observed in the case of PM_10_ and PM_100_ fractions. However, taking into account similar orders of magnitude, both values can be considered representative.

**Figure 4:**
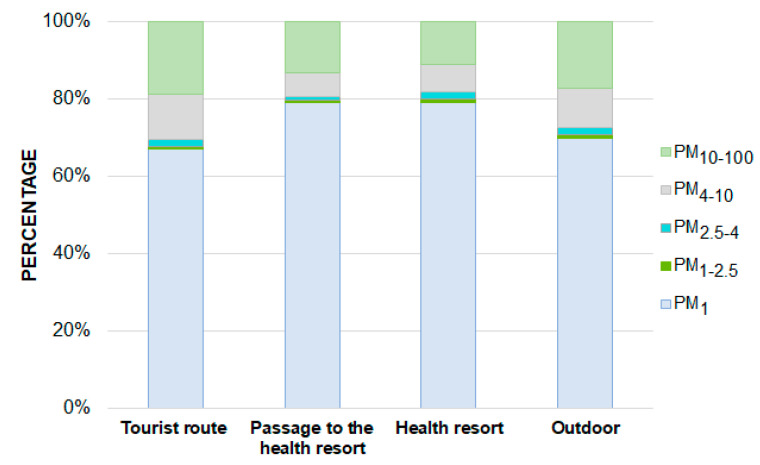
Average percentages of individual PM fractions in the total mass of PM on the tourist route, in the passage to the health resort, in the health resort and outside the mine.

**Figure 5:**
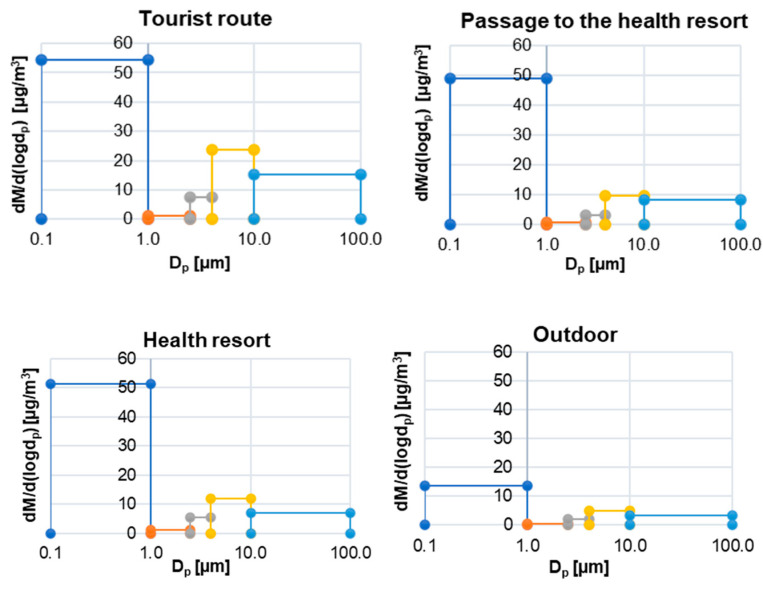
PM mass size distribution on the tourist route, in the passage to the health resort, in the health resort and outside the mine.

Figure 4 presents the percentages with respect to the aerodynamic diameter of particles in the total mass of PM at individual measurement points. On average, in each of the considered mine locations, most of the PM mass was accumulated in particles with a diameter in the range of 0.1–2.5 µm (68–80%). The highest average share of fine particles in the total mass of PM was recorded in the passage to the health resort and in the health resort (80% in both places). At all measurement sites, the percentage of particles with diameters below 2.5 µm in the total mass of PM was relatively constant with a slight increase accompanying the increase in coarse PM at the times described above (passing tours, restaurants, large spaces) (Figure 3). The high contribution of fine PM in the total PM mass is also evidenced by the low MMAD values, which means that on the tourist route 50% of the PM mass is concentrated in particles with diameters below 2.49 µm, while in the passage to the health resort and in the health resort, these diameters are respectively 1.48 µm and 1.46 µm (Table 3). It is worth adding that the desired particle diameter for effective inhalation therapy is in the range of 1–3 µm [47]. The PM mass size distributions in all of the considered locations were similar—they were bimodal and their grater modes were between 0.1–1 µm while the smaller ones were between 4–10 µm (Figure 5). In the passage to the health resort and in the health resort, the lowest share of particles with diameters ranging from 10–100 µm was recorded. It was probably because the number of people entering these rooms is small in relation to the number of people visiting the tourist route. As shown above, in the summer months, patients constitute <2% of visitors to the mine [32], therefore the turnover of people in the health resort is much lower than on the tourist route. In addition, there are no tourist attractions in the passage to the health resort where visitors can stop and visit. In addition, there are no tourist attractions in the transition to the spa where you can stop and visit. Each patient spends at least 2.5 hours a day in this place [48]. On the tourist route, traffic is continuous. Considering the changes in the shares of individual PM fractions, attention should be paid to the relatively constant share of fine PM. The fact that the level is constant (approx. 50 µg/m^3^) in almost every point of the tourist route, in the passage to the health resort and in the health resort, may indicate its internal origin. Taking into account the results of previous studies on the composition of PM of the salt mine in Wieliczka [10,11], with high probability it can be assumed that the source of these particles is the structural material overlying the walls of the tunnels and these particles contain significant amounts of NaCl, which has a beneficial effect for human health. There are several reasons why the influence of the atmospheric air on the recorded PM concentrations can be excluded. First, there are ventilation solutions. Although the air entering the mine from the outside contains pollutants of anthropogenic origin, during the airflow through the mine’s ventilation tunnel system it is cleaned and the concentrations of these pollutants are trace [10]. Mechanically generated air movement can affect the efficient air exchange, and thus the effective removal of pollutants from the environment, especially coarse particles [49]. The method of connecting selected chambers and corridors also enables their systematic ventilation. Secondly, the main construction material of the walls of tunnels and chambers, containing primarily NaCl, absorbs the atmospheric air and anthropogenic pollutants [10]. Another factor contributing to limiting the concentration of coarse PM in the mine is high air humidity, which, according to measurements carried out systematically by mine employees, ranges from 60–75% [50]. The high humidity resulted in a low degree of resuspension, therefore a small proportion of coarse PM [51,52].

The PM concentrations described in this manuscript are higher than the average mass concentration of TSP measured in two health resort chambers of the same salt mine (Eastern Mountains Stable Chamber, Dragon Chamber) in July 2015 by Rogula-Kozłowska and Badyda with their teams (30 µg/m^3^) [10]. In addition, the recorded results are higher than the average TSP concentration measured in the salt mine in Bochnia by Puławska and her team in June 2019 [15], where for 6 measuring points located in underground chambers it was 38.4 µg/m^3^, and the average concentration of PM_4_ was approx. 12 µg/m^3^. The obtained results were also compared with the results of the tests carried out in the salt mine in Wieliczka in 1992 [53], according to which the average concentrations of fine and coarse PM were in the range of 20.4–123.0 µg/m^3^ depending on the measuring point (St Anthony’s Chapel and St Kinga Chapel). Probably, the differences in the results are due to differences in the location of the measurement points (compared to previous studies in the same mine), differences in the mine’s characteristics (compared to the Bochnia mine), and different methods used in the measurements. The PM concentrations described in the above-mentioned publications were measured using the gravimetric method. Moreover, in the first part of the study [10], TSP and PM_4_ concentrations were carried out at the inlet and outlet, where there is intensive ventilation. In addition, optical meters are sensitive to high humidity. In the case of high humidity, PM concentrations may be overstated, as fine drops of water vapour are identified by optical devices as PM particles [36]. The problem of overestimating the results by optical meters was also highlighted in [54,55,56]. To avoid errors related to the use of the optical meter, as described in Section 2.2, the meters used in the studies were calibrated and tests for equivalence with the reference method were performed. The results obtained with the DustTrak DRX 8534 meter were converted based on the obtained linear regression coefficient (a). In the future, when using optical meters, it is worth using air dryers for such measurements, e.g., those used in the studies described in [57]. However, the optical method was used to show the influence of tourist traffic and the activity of residents on PM concentration.

As shown in Section 2.2, together with the measurements of the concentrations of the five PM fractions inside the mine, the concentrations of the same PM fractions outside were measured in parallel. During the research, the weather conditions outside were stable. Measurements at the meteorological station in Wieliczka showed an average air temperature of 22.3 °C, mainly west wind with an average speed of about 2.8 m/s and slight showers of rain, mainly in the afternoons. All measurements were made during the summer months. In this way, the potential impact of seasonal fluctuations on PM concentrations was minimised. The average concentrations of PM_1_, PM_2.5_, PM_4_, PM_10_ and PM_100_ in the atmospheric air ranged from 13.7–19.6 µg/m^3^ and they were more than three times lower than the average concentrations of the same PM fractions on the tourist route of the mine or in the health resort (Table 2, Figure 3). PM in the atmospheric air contained mainly ultrafine and fine particles, which accounted for approx. 70% of the total PM mass (Figure 4). Compared to the PM concentrations in the mine, no significant fluctuations in PM concentrations were noted during the measurements. Small fluctuations in PM concentrations noted between 33′ and 36′, in 44′, in 1 h 40′ of the measurement, were probably caused by the movement of security personnel moving near the device. The average concentrations of PM outside the mine are typical concentrations for this period and urban areas in Poland, not indicating the impact of additional sources in the immediate vicinity of the mine [25,58,59,60,61,62,63]. As the measurements were carried out using the same measurement methods and the meters were calibrated in relation to the gravimetric method as well as in relation to each other, it can be confirmed that high concentrations of PM in underground measurement points are mainly determined by the aerosol of internal origin and exclude the influence of external sources

### 3.2. Particulate Matter Deposition and Dose of Dry Salt Aerosol Inhaled during the Stay in the Mine

Total PM deposition factors (DF) for all analysed measuring points in the mine for both age groups take values in the range 0.58–0.70 (Table 4). This means that during a stay in a mine/health resort, 58 to 70% of the inhaled PM is deposited in the respiratory tract during one respiratory cycle. According to Newman’s research, saline inhalations with the use of nebulisers result in the deposition of 10–40% of the inhaled substance (physiological saline solution of 0.9% NaCl) in the lungs, and the effectiveness of inhalation depends, among others, on skills, mask tightness and equipment efficiency [64,65]. The literature review proves that the studies described in this publication are one of the first studies on PM deposition in the respiratory tract of people receiving therapy in mine chambers, therefore the authors compared the obtained results to the results of studies carried out in indoor sports rooms, where a similar deposition model was used [66]. Comparing the results of these two studies, it can be concluded that the DF in the mine is higher than the DF calculated for indoor sports rooms, where for people at rest they take values in the range 0.32–0.35, and 0.50–0.65 for exercising people, assuming twice the frequency and depth of breathing caused by physical activity [66]. Relatively high DFs in the mine result from the high percentage of fine particles in the PM mass. Regarding Table 4, DF estimated for children is greater than for adults, which is closely related to the difference in their lung morphometry [40]. In adults over 21 years of age, most of the inhaled PM is deposited in the upper respiratory tract, while in children more than 50% of the PM mass reaches the alveoli. It should also be noted that patients staying in the health resort often participate in gymnastic classes. During exercise, the frequency and depth of breathing increase, therefore, for those exercising, the deposition rates may be even greater than those estimated [67]. Based on the results of previous studies, which showed that NaCI constitutes 88% of the PM mass [10], in addition to DF, Table 4 presents the average doses of therapeutic dry salt aerosol inhaled by a tourist/patient. Often people going to the health resort first visit the mine. In such cases, the three doses calculated for the tourist route, passage to the health resort and health resort should be added together. The highest dose of dry aerosol (NaCl) is inhaled during the stay in the health resort, which results from the longest time spent there (Table 4). During a single stay in this place, an adult takes about 43 µg of dry salt aerosol and a child about 24 µg. The difference between these two groups is due to different inhalation rates (Table 1), depending on age. Aerosol deposition and thus the doses of the inhaled aerosol may differ from one person to another, partly because of random variations in airway geometry which affect the probabilities of impaction and sedimentation of salt aerosol. In addition, deposition may be slightly different in patients with asthma or chronic bronchitis whose airways may be constricted by swelling and excessive mucus secretion.

It is difficult to compare the obtained results with other studies due to the lack of evidence in this area. The research conducted so far has focused mainly on the study of the concentration of salt aerosol and the calculation of the absorbed dose of moist aerosol [8]. Also due to other measurement techniques, it is difficult to relate the obtained values to those described in other publications. Nevertheless, taking into account the fact that the concentrations of moist salt aerosol in the more and more common ground salt caves are many times lower (0.45 mg/m^3^) than in the salt mine chambers in Wieliczka (22 mg/m^3^) [8], it can be assumed that also the doses of dry NaCl inhaled in these places will be lower.

Taking into account the development of PM prediction systems [68,69,70], this work may constitute an introduction/premise for the creation of a large database on PM concentrations and composition, e.g., in underground health resorts, salt caves, seaside resorts and other similar places. As these places offer various variants of stays (1-, 3-, 14-day stays, etc.), prognostic models based on the concentration of PM and its chemical composition (mainly NaCl content) could be used to estimate the benefits of staying in underground health resorts or salt caves in various time variants. They can also be applied to determine the optimal variants of the duration of a therapeutic stay for different age groups, indicating the points of the mine and health resort where it is most advantageous to conduct treatments, or comparisons of inhaled NaCl doses during a stay in underground chambers or salt caves with inhaled NaCl doses, e.g., in a coastal environment.

## 4. Conclusions

The research assessing the concentrations of five PM fractions (PM_1_, PM_2.5_, PM_4_, PM_10_ and PM_100_) on the tourist route of the salt mine in Wieliczka, in the passage to the health resort, in the health resort and outside of the building provides the following conclusions:

- In general, the highest concentrations of PM were recorded on the tourist route (54.5–81.2 µg/m^3^), while the lowest in the passage to the health resort (48.9–61.7 µg/m^3^).
- At all measuring points, the vast majority of the PM mass (more than 60%) was accumulated in fine particles with aerodynamic diameters smaller than 2.5 µm.
- The highest concentrations of PM were recorded in places where tourists were passed and in chambers characterised by large cubature, as well as in places with large rock walls.
- Tourist traffic undoubtedly affects the concentration of coarse PM in the underground chambers, however, the microclimate of the mine and ventilation solutions cause a quick and effective reduction of the concentration of this PM fraction.
- High air humidity in underground chambers prevents PM resuspension.
- Probably due to the relatively short time of measurements, PM concentrations outside the mine were at a relatively constant level. The comparison of the distribution of PM concentrations inside and outside the mine allows us to exclude an influence of atmospheric air and anthropogenic sources on PM concentrations inside the mine.
- In this manuscript, the concentrations of the dry aerosol with particles smaller than 100 µm were examined. The wet aerosol concentrations in the mine are certainly higher.
- Due to the high air humidity in the underground chambers of the mine, in the future, when measuring PM concentrations in real-time with the use of optical meters, it is worth using solutions allowing for air drying.

Assessment of PM deposition and inhaled dry salt aerosol dose during the stay in the mine provides the following conclusions:

- Due to the high proportion of fine particles in the total PM mass, the PM deposition factors in the respiratory tracts of patients and mine visitors are relatively high (0.58–0.70).
- High PM deposition factors at all measurement points and prevalence of respirable particles in PM composition guarantees the efficiency of action and penetration of all sections of the respiratory tract right up to the deepest, which at the same time indicates the high therapeutic effectiveness of the stay in the mine, even while visiting the mine.
- In children, most of the PM is deposited in the deeper parts of the respiratory system (trachea and bronchi and alveoli), while in adults the largest mass of dry NaCl is deposited in the upper respiratory tract.
- During a single visit to the mine’s tourist route, going to the health resort and treatment in the health resort, an average adult inhales approximately 84 µg of dry salt aerosol.

There are few publications on PM concentrations in mines/underground health resorts and the possibilities of comparing the obtained results are limited. The authors believe that the present study makes an important contribution to the limited knowledge on the pulmonary effects of a subterranean microclimate. Moreover, the presented research may constitute an introduction to further and broader studies on the distribution of PM concentrations and its deposition, e.g., in more and more popular ground-based salt caves or ground-based health resorts.

## Figures and Tables

**Table 1:** Physiological parameters implemented into MPPD model and estimating the average dose of inhaled aerosol.

Age	Respiration Frequency [Breath/min]	Breathing Volume [mL]	Lung Volume [mL]	Upper Airway Volume [mL]	InhR [m^3^/day]
Children 8+ years	17	278.20	740.42	21.03	7.63
Adults 21+ years	14	477.20	2792.50	42.30	15.20

**Table 2:** Descriptive statistics of PM_1_, PM_2.5_, PM_4_, PM_10_ and PM_100_ on the tourist route, in the passage to the health resort, in the health resort and outside the mine.

Measurement Point	PM Fraction	Statistical Parameter				
		Mean [µg/m^3^]	Median [µg/m^3^]	Minimum [µg/m^3^]	Maximum [µg/m^3^]	Standard Deviation
Tourist route	PM_1_	54.5	53.9	44.2	155.0	7.6
	PM_2.5_	55.0	54.5	44.3	156.0	7.9
	PM_4_	56.5	56.1	44.4	158.0	8.9
	PM_10_	66.0	65.0	44.8	177.0	16.1
	PM_100_	81.2	78.1	45.5	288.0	28.5
Passage to the health resort	PM_1_	48.9	48.3	44.0	73.5	3.5
	PM_2.5_	49.1	48.5	44.1	74.5	3.6
	PM_4_	49.7	49.1	44.2	76.0	3.9
	PM_10_	53.5	52.9	44.3	85.9	5.2
	PM_100_	61.7	60.9	44.3	101.0	9.5
Health resort	PM_1_	51.4	50.2	43.9	117.0	5.7
	PM_2.5_	51.9	50.7	44.0	118.0	6
	PM_4_	53.0	51.6	44.0	121.0	6.8
	PM_10_	57.7	54.4	44.3	152.0	11.1
	PM_100_	64.8	59.4	44.3	222.0	19.9
Outdoor	PM_1_	13.7	11.0	7.0	73.0	8.3
	PM_2.5_	13.9	11.0	7.0	73.0	8.3
	PM_4_	14.3	11.0	8.0	73.0	8.3
	PM_10_	16.3	13.0	8.0	73.0	9.2
	PM_100_	19.6	15.0	8.0	75.0	12.8

**Table 3:** Mass median aerodynamic diameter (MMAD) and geometric standard deviation (GSD) of particle mass size distributions (PSD) on the tourist route, in the passage to the health resort, in the health resort and outside the mine.

Place of Measurement	MMAD [µm]	GSD [µm]
Tourist route	2.49	10.38
Passage to the health resort	1.48	9.44
Health resort	1.46	8.75
Outdoor	2.19	9.98

**Table 4:** Particulate matter deposition factors (DF) in the three regions of the respiratory tract (H—upper respiratory tract, TB—trachea and bronchi, P—alveoli) of the two age groups and average doses of dry salt aerosol inhaled during the stay at individual points of the mine.

Sampling Site	DF						Total DF		t [h]	Dose [µg]	
	Adults			Children			Adults	Children		Adults	Children
	H	TB	P	H	TB	P					
Tourist route	0.43	0.05	0.11	0.33	0.17	0.17	0.59	0.67	2.00	37.16	21.18
Passage to the health resort	0.34	0.05	0.19	0.3	0.09	0.31	0.58	0.70	0.25	4.02	2.43
Health resort	0.33	0.05	0.2	0.26	0.08	0.31	0.58	0.65	2.50	42.83	24.09

## Data Availability

Not applicable.
